# Covered metallic stent for the treatment of malignant esophageal fistula combined with stricture

**DOI:** 10.1186/s12876-020-01398-6

**Published:** 2020-07-30

**Authors:** Yonghua Bi, Mengfei Yi, Zepeng Yu, Xinwei Han, Jianzhuang Ren

**Affiliations:** Department of Interventional Radiology, The First Affiliated Hospital of Zhengzhou University, No.1, East Jian She Road, Zhengzhou, 450052 China

**Keywords:** Esophageal fistula, Esophageal stents, Drainage, Esophageal strictures

## Abstract

**Background:**

Esophageal fistula and stricture is rare but life-threatening complication for esophageal cancer. The management of esophageal fistula and stricture remains challenging. We aimed to determine the safety, feasibility and efficacy of covered metallic stent and three tubes placement for the management of esophageal fistula and stricture.

**Methods:**

Between May 2012 and March 2018, all patients with esophageal fistula and stricture were treated using three tubes or covered metallic stent placement. Patients in group A received covered stents and three tubes placement. Patients in group B only received three tubes placement. Continue abscess drainage and nutritional support was performed after procedure. Three tubes or esophageal stents were removed once esophageal fistula heals. The related medical records were retrospectively assessed.

**Results:**

Thirty-seven consecutive patients with esophageal fistula and stricture were enrolled, including 26 patients in group A and 11 patients in group B. Stent placement procedure was technically successful in 25 patients (96.2%). A total of 42 covered stents were inserted. Seventeen esophageal stents were successfully removed from 10 patients. The median retention duration was 3.3 months and 3.4 months for stent and abscess drainage tubes, respectively. One perioperative death due to massive hemorrhage was observed 21 days after stent placement. The abscess cavity was decreased or disappeared in 17 cases and 4 cases in group A and group B, respectively. During follow up, patients in group A still showed a significant better condition of normal diet than that in group B (*p* < 0.05). Fourteen patients died of cancer recurrence, 3 patients died of massive digestive bleeding and 2 patients died of severe pulmonary infection. The median survivals were 14.8 months and 13.2 months for group A and group B, respectively.

**Conclusions:**

Covered metallic stent placement is safe, feasible and efficacious for treatment of esophageal fistula and stricture, with a better condition of normal diet than patients only received three tubes placement.

## Background

Esophageal fistula combined with esophageal stricture is a rare but life-threatening complication for patients with esophageal cancer or esophagogastric carcinoma [1, 2]. Contamination of abscess cavity may cause massive digestive bleeding or even death. Most of patients with esophageal carcinoma will receive only palliative treatment, considering that those patients often show advanced inoperable disease at the time of diagnosis.

Various conservative protocols have been reported for the management of esophageal fistula, including biodegradable fistulae plugs, transluminal drainage and esophageal stent placement [3–6]. Plain malignant esophageal strictures can be successfully treated and show a satisfactory result. However, in the presence of an esophageal fistula, palliation presents a challenge and has a poor prognosis [2, 4, 7]. There are few reports of studies on the treatment for esophageal stricture complicated with fistula [8]. In this study, three tubes and covered esophageal stent was used. We aimed to determine the safety, feasibility and efficacy of this method for the management of esophageal fistula and stricture.

## Methods

### Patient selection

**Fig. 1:**
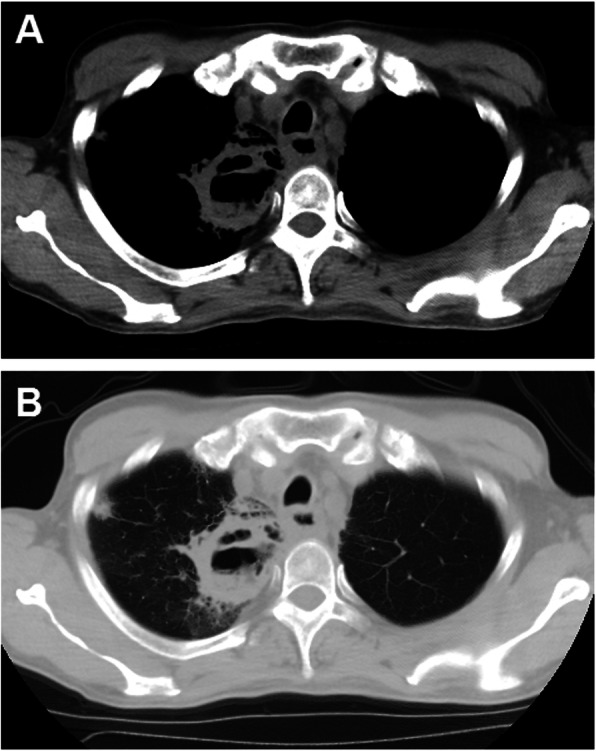
Chest CT scans for 66-year-old man with esophageal fistula and stricture. Chest CT scan in the mediastinal (**a**) and lung windows (**b**) show mediastinal abscess without obvious pleural effusion before procedure

**Fig. 2:**
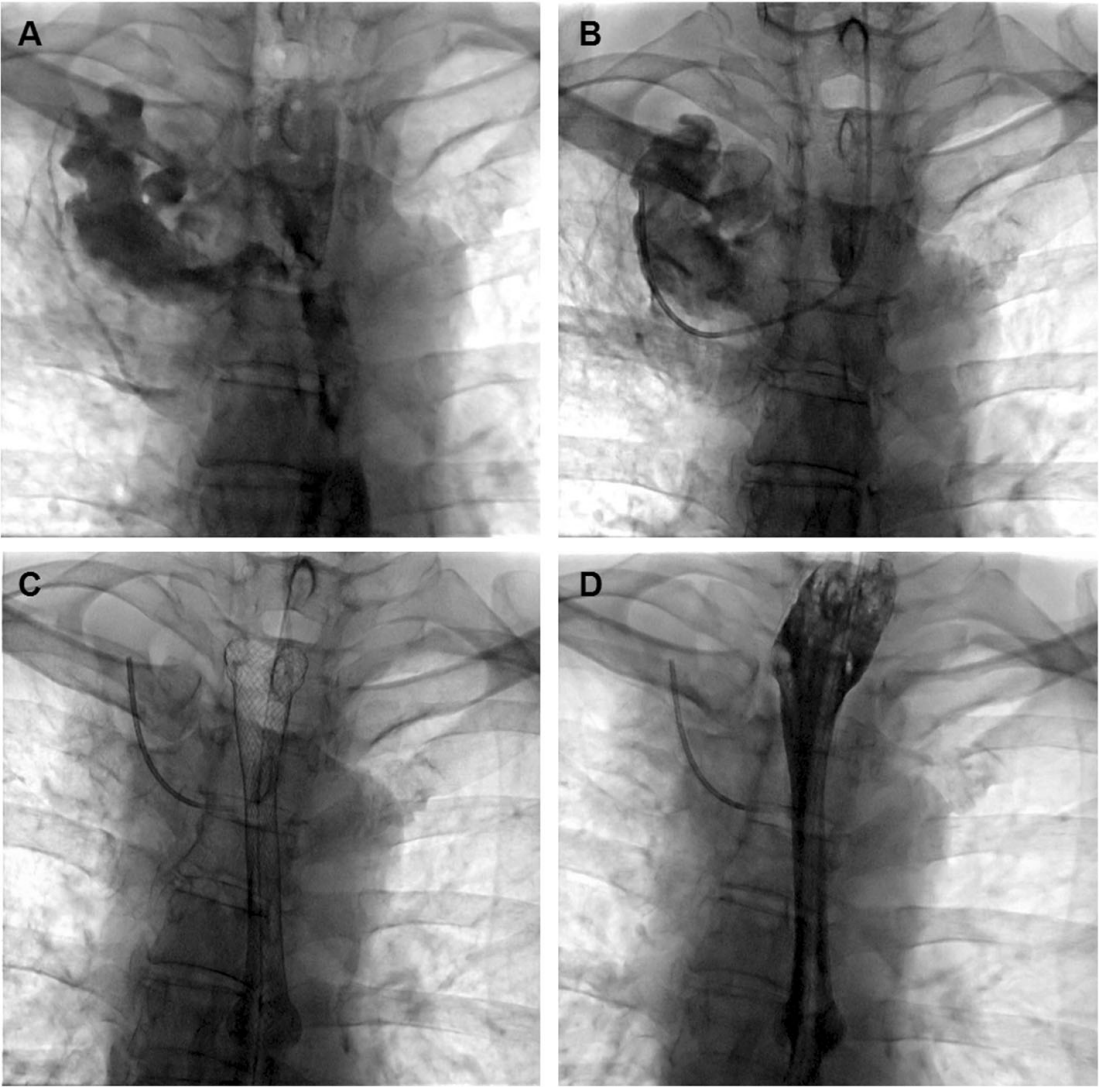
Three tubes and covered stent placement. **a** Esophagography shows esophageal mediastinal fistula and esophageal stricture in the middle part of esophagus. **b** Abscess drainage tube was inserted. **c** An esophageal covered stent was placed to block the fistula. **d** Esophagography shows that the contrast agent flows though the stent with no fistula immediately after stent placement

This study was approved by the Ethics Committee Board of First Affiliated Hospital of Zhengzhou University. Informed consent was obtained from each patient. Between May 2012 and March 2018, all patients with malignant esophageal fistula and stricture in our institution were enrolled and retrospectively analyzed. The diagnosis of an esophageal fistula and stricture was made according to the finding of chest computed tomography (Fig. 1) and esophagography (Fig. 2a). According to the treatment protocol, patients were divided into two groups. All patients were suitable for placement of covered stents and three tubes placement (group A), however, some patients were unable to receive stents placement because of economic pressure or reluctance to receive stent implantation and only received three tubes placement (group B). The three tubes are presumably a nasocavity drain placed fluoroscopically, a nasojejunal feeding tube and a nasogastric drainage tube, this should be stated clearly. After normal eating, the three tubes and stents were removed. Patients in group A whose fistula has disappeared by esophagogram can start normal diet 3–5 days after stent placement. Patients in group B are not advised to eat through mouth unless the fistula has disappeared, so as not to aggravate the infection.

### Three tubes placement

All procedures were performed under fluoroscopic guidance. The esophagus was anesthetized by taking lidocaine gel. A 5-F catheter was introduced into the distal end of abscess cavity, followed by exchange with a 5-F pigtail catheter or 5-F straight catheter (Cook Medical, Inc., Bloomington, IN). The abscess cavity was rinsed with saline daily, and continuous negative pressure suction was performed to make sure effective drainage (Fig. 2b). A jejunal feeding tube was placed for infusing enteral nutrition solution. The gastrointestinal decompression tube was inserted to decrease reflux. Patients did not feed by mouth until complete block of fistula by covered stent or successful sealing was confirmed via esophagography.

### Covered esophageal stent placement

The self-expandable metallic stents was covered by polytetrafluoroethylene membrane (Nanjing Micro-Tech Medical Company, Nanjing, China). The stent was made of nitinol alloy and the outside of the stent were covered. Stent diameter ranges from 14 to 22 mm (interval 2 mm) and stent length ranges from 60 to 160 mm (interval 20 mm). Recovery lines in the proximal end are used for the adjustment or recovery of the stent. The delivery system of stent is 8 mm in diameter and 650 mm in length.

**Fig. 3:**
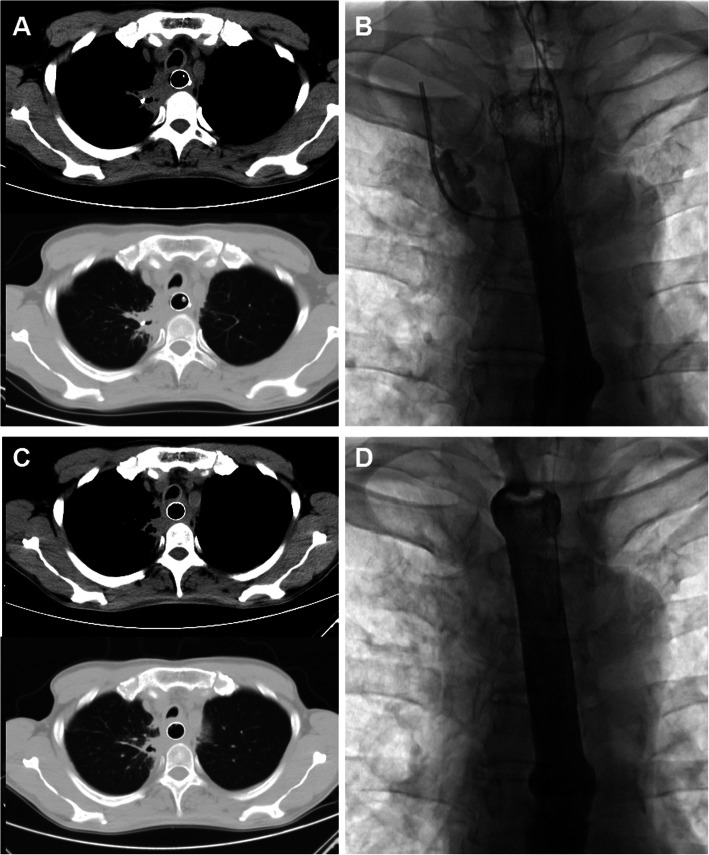
Examinations after stent placement during follow up. **a** The chest CT scan shows decrease of mediastinal abscess with no pleural effusion 2 month after procedure. **b** Esophagography shows decrease of esophageal mediastinal abscess, and then the drainage tube was removed 2 months after procedure. **c–d** The chest CT scan and esophagography shows almost disappearance of mediastinal abscess 2.7 months after stent placement

A 5-F cobra catheter was inserted into the gastric cavity, followed by exchange with a stiff guide wire. Along the stiff guide wire, a covered stent system was delivered and released to block the esophageal fistula. A repeated esophagography was performed immediately after stent placement to confirm the closure of fistula (Fig. 2c-d). During follow up, chest computerized tomography (CT) and/or esophagography were performed to show the change of the abscess cavity. The three tubes were removed if abscess cavity disappear was confirmed by esophagography and chest CT (Fig. 3). The indications for stent removal include severe restenosis leading to dysphagia, obvious stent migration leading to failure of stent adjustment, healing of fistula and stenosis relief to return to normal diet.

### Statistical analysis

Data were expressed as mean ± standard deviation or median ± interquartile range (IQR), and analyzed by student t test. Qualitative data were expressed in percentage, and data between Group A and Group B were analyzed by Fisher’s exact test. Patency rate was compared by Log-rank (Mantel-Cox) Test (GraphPad Software, Inc., USA). Statistical significance was considered when *p* < 0.05.

## Results

### General information

A total of 37 patients with esophageal fistula combined with stricture were enrolled in this study, including 26 patients in group A and 11 patients in group B.. The ages of the patients ranged from 43 years to 77 years, for a median age of 62.0 years. Twenty-three patients showed a fever, for a median temperature of 38.6 °C (IQR: 38.4 to 39.0 °C). The median course of disease before referral to our department was 6.0 months (IQR: 3.0 to 17.5 months). The median interval between clinical symptom and diagnosis of fistula was 2.0 months (IQR: 1.0–5.0 months). The median interval between diagnosis of fistula and interventional treatment was 0.5 months (IQR: 0.2–3.0 months). There were 23 cases of esophageal mediastinal fistula and 14 cases of esophagopleural fistula. All patients showed esophageal stricture simultaneously. Twenty-six patients received stents and three tubes placement in group A, and 11 patients received three tubes placement in group B. There was no significant difference in characteristic data between two groups before interventional treatment (all *p* > 0.05, Table 1).

**Table 1:** Patient characteristics before interventional treatment

	Total	group A	group B	*P*
n	37	26	11	
Gender, Male	31 (83.8%)	21 (80.8%)	10 (90.9%)	0.646
Median age (years)	62.0 (54.5, 66)	63.0 (54.8, 65.3)	59.0 (53, 69)	0.702
Esophageal mediastinal fistula	23 (62.2%)	14 (53.8%)	9 (81.8%)	0.150
Esophagopleural fistula	14 (37.8%)	12 (46.2%)	2 (18.2%)	0.150
Disease course before referral to hospital	6.0 (3.0, 17.5)	6.0 (3.0, 16.0)	9.0 (3.8, 63.5)	0.221
Interval between symptom and diagnosis	2.0 (1.0, 5.0)	2.0 (0.5, 5.4)	3.0 (1.0, 5.0)	0.133
Interval between diagnosis and treatment	0.5 (0.2, 3.0)	0.5 (0.2, 3.0)	0.7 (0.1, 1.8)	0.329
Patients with normal temperature	13 (35.1%)	9 (34.6%)	4 (36.4%)	1.000
Highest temperature of fever patients	38.6 (38.4, 39.0)	38.6 (38.2, 39.0)	38.7 (38.4, 39.0)	0.981
Highest leukocytes count (×10^9^/L)	9.2 (6.6, 12.3)	8.9 (5.8, 11.6)	11.3 (7.7, 18.0)	0.831
Highest neutrophil (%)	82.4 (75.1, 89.0)	81.5 (68.2, 88.4)	83.1 (79.3, 92.0)	0.103

Besides, eleven patients received chemotherapy or radiotherapy, and six patients received chemotherapy and radiotherapy. For patients with squamous cell carcinoma and HER-2 negative adenocarcinomas, fluorouracil + cisplatin, or paclitaxel/docetaxel + cisplatin/nedaplatin were used. Patients with HER-2 positive adenocarcinoma were treated with trastuzumab combined with fluorouracil + cisplatin. Radiotherapy was performed for 2–30 times for each patient. One patient with metastatic tumor in lung received iodine-125 seeds implantation therapy.

### Interventional procedure outcomes

Three tubes were placed successfully for all patients (100%). One patient failed in stent placement owing to complete occlusion, for a technical success rate of 96.2% (25/26). A total of 42 covered esophageal stents were placed in group A, for a median diameter of 18 mm (IQR: 18–20 mm), median length of 120 mm (IQR: 100–120 mm). Only one stent was placed in each of the 16 patients. For the remaining 10 patients, two or more stents were used due to stent restenosis or migration. Stent insertion was technically successful in the remained 25 patients, with satisfactory expansion and appropriate position. Complete block of esophageal fistula was confirmed by esophagography in 24 patients immediately after covered stent placement, and one patient showed a small amount of fistula between the stents and the wall of the esophagus. There was no significant difference between group A and group B regarding average times of admissions, mean hospitalization days, or total hospitalization days. However, at the time of discharge, 6 patients were fed with nutrition tube and 20 patients were fed a normal diet in group A. However, 9 patients were fed with nutrition tube in group B, which was significantly lower rate of normal diet than that in group A (*p* = 0.0023, Table 2). Seventeen esophageal stents were successfully removed from 10 patients due to stent restenosis (*n* = 9), migration (*n* = 6), and healing of fistula and stenosis relief (*n* = 2), with no major complications. The median retention duration was 3.3 months (IQR:1.8, 7.0 months) and 3.4 months (IQR:1.4, 5.0 months) for stent and abscess drainage tubes, respectively.

**Table 2:** Clinical outcomes after interventional treatment

	Total	group A	group B
Average times of admissions	2.0 (1.0, 4.5)	2.0 (1.0, 4.3)	3.0 (1.0, 5.0)
Mean hospitalization days	17.0 (9.3, 21.0)	17.0 (8.7, 22.1)	11.8 (9.5, 21.0)
Total hospitalization days	37.0 (21.0, 75.4)	38.5 (21.8, 87.8)	33.9 (18.0, 70.8)
Dysphagia score before treatment	3.5 (3.0, 4.0)	3.0 (3.0, 4.0)	4.0 (3.0, 4.0)
Dysphagia score during follow up	0.0 (0.0, 3.0)	0.0 (0.0, 0.8)	3.0 (0.0, 4.0) ^a^
Normal diet when discharge	22 (59.5%)	20 (76.9%)	2 (18.2%) ^a^
Normal diet during follow up	28 (75.7%)	22 (84.6%)	6 (54.5%) ^a^
Decrease/disappear in abscess cavity	21 (56.8%)	17 (65.4%)	4 (36.4%)
Duration for abscess cavity decrease	114.5 (67.0, 214.5)	173.0 (108.0, 337.0)	93.5 (53.5, 185.5)
Median survival (months)	13.2	14.8	13.2

^a^group A vs group B, *p* < 0.05

### Complications

No esophagus perforation was observed during procedure. One perioperative death due to massive hemorrhage was observed 21 days after stent placement. Except for one perioperative death, two patients died of massive digestive bleeding in group A during follow up. Stent migration was found in 8 patients, for a migration rate of 30.8% (8/26). The migrated stent was adjusted by using string. Five patients showed stent restenosis, for a restenosis rate of 19.2% (5/26). All migrated or stenotic stents were adjusted or replaced for 0 to 3 times (median: 1 time). Occlusion of abscess drainage tube was found in one patient. The abscess drainage tube was adjusted or replaced for 0 to 7 times (median: 1 time). One patient showed slight fistula due to poor adhesion after stent placement (Table 3).

**Table 3:** Complications and follow up

	group A	group B
Complications, n (%)		
Occlusion of abscess drainage tube	0 (0.0%)	1 (9.1%)
Stent migration	8 (30.8%)	–
Stent restenosis	5 (19.2%)	–
Fistula due to poor adhesion	1 (3.8%)	–
Perioperative death	1 (3.8%)	
Loss to follow up	2 (7.7%)	0 (0.0%)
Survival without severe symptom	11 (42.3%)	5 (45.5%)
Death cause, n (%)		
Cancer recurrence	9 (34.6%)	5 (45.5%)
Massive digestive bleeding	3 (11.5%)	0 (0.0%)
Died of pulmonary infection	1 (3.8%)	1 (9.1%)

### Follow-up

Thirty-five patients were successfully followed up, and 2 patients were lost to follow up in group A. Chest CT showed that the abscess cavity was decreased or disappeared after a median duration of 114.5 days in 17 cases in group A, 4 cases showed decreased or disappeared abscess cavity after a median duration of 173.0 days in group B. At the day discharge, 20 patients showed normal diet per os in group A, and only 2 patients showed normal diet in group B. During follow up, patients in group A still showed a significant better condition of normal diet and lower dysphagia score than that in group B (*p* < 0.05). At this time, 11 patients in group A were still alive without severe symptom, and 5 patients in group B return to normal living conditions with no severe symptom. During follow up, 14 patients died of cancer recurrence, 3 patients died of massive digestive bleeding and 2 patients died of severe pulmonary infection. The median survivals were 14.8 months and 13.2 months for group A and group B, respectively.

## Discussion

Malignant esophageal fistula combined with stricture can be found in patients with esophageal or esophagogastric carcinoma or anastomotic leakage/stricture after esophagectomy [1, 2]. Palliation presents a challenge with a poor prognosis and relatively high mortality [2, 4, 7–11]. Unfortunately, there is no optimal treatment protocol for those patients [2–7, 12]. Esophageal stents have been served as a palliative treatment for patients with esophageal diseases [13–19]. Management of esophageal fistula needs adequate elimination of contamination and effective drainage of the abscess cavity.

Covered esophageal stent placement and abscess drainage tube insertion was used in this study. Our data indicated that fluoroscopically guided placement of metallic covered stents or three tubes are safe. After covered stent placement, the abscess cavity is also allowed to continuously drainage by abscess drainage tube. According to our experience, the esophageal stent placed in the stricture site often show incomplete expansion, and show complete expansion and can close the fistula about 3–5 days later. We recommend patients received nasal feeding nutrition after stents placement, and start normal diet if the fistula disappears. All patients were suitable for placement of covered stents and three tubes placement. Unfortunately, patients in group B did not receive stents placement because of economic pressure or reluctance to receive stent implantation. Those patients only received three tubes placement, and showed a worse condition of normal diet.

However, covered stent also bring certain related complications. All stents used in our study was covered, and stent migration was a common complication. Eight patients (30.8%) showed stent migration and 5 patients showed stent restenosis (19.2%). In other studies, stent migration rates ranged up to 19% for plastic covered stents and 22.7% for segmental covered stents [20, 21]. Compared with radioactive metal stent, our study showed a higher restenosis rate, indicating that radioactive metal stent may prevent stent restenosis induced by tumor progression [20].

There were some limitations in the current study. This was a retrospective study and the enrolled patient number was relatively small. Patients were divided into different groups according to their choice based on doctors’ advice, treatment willingness and economic endurance. A prospective randomized study is needed to reach a positive and convincing clinical conclusion.

## Conclusions

Covered metallic stent placement is safe, feasible and efficacious for treatment of esophageal fistula and stricture, with a better condition of normal diet than patients only received three tubes placement.

## Data Availability

For further details, the corresponding author can be contacted.

## Abbreviations

CT: Computerized tomography
IQR: Mean ± interquartile range
